# Invalid Children

**Published:** 1904-06-04

**Authors:** 


### The Hospital.

A Journal of

The Medical Sciences and Hospital Administration.

Vol. XXXVI.—No. 923. JUNE 4, 1904.

### INVALID CHILDREN.

Pending the arrival of the time when Mr. Galton and his followers, by means of the diligent prosecution and practice of the new science of "Eugenics," will have succeeded in relieving the world of its present grievous burden of sickly or crippled children, every device by which that burden can be lightened is at least worthy of serious consideration; and we have therefore much pleasure in calling the attention of our readers to the Conference which will be held at the Guildhall, under the auspices of the Invalid Children's Aid Association, on the 7th and 8th instant. The morning meetings of the respective days will be under the chairmanship of the Earl of Aberdeen and of the Duchess of Sutherland; the afternoon meetings under that of Sir William Broadbent and of Lord Stanley of Alderley; and at all four meetings, papers on important questions will be read by persons entitled to speak with authority in relation to them. Thus on Tuesday morning, the 7th, a paper is promised by Mr. Warrington Haward, F.R.C.S., on the past work of the Association, and another by Dr. Newsholme, of Brighton, on "The Social Aspects of the Prevention of Illness." In the afternoon Dr. Cautley will deal with "Cow's Milk as a Food in Infancy," Dr. Raw with "The Prevention of Tuberculosis among Children," and Messrs. Evans and Tubby will read papers on "Joint Disease and Surgical Tuberculosis." On Wednesday the Duchess of Sutherland will describe the work of the Cripples' Guild in the Potteries, and her paper will be followed by others of a practical kind on the apprenticeship and the industries of cripples; while the afternoon session will be devoted to questions concerning the school education of the physically or intellectually defective. It will be seen that the work of the Conference covers a wide area, and that many of the questions which it is proposed to discuss are of considerable national importance. We are sorry not to see the name of Mrs. Humphry Ward in the programme of business, but trust that her large experience in relation to the school training of cripples will not be withheld from the assembly.

At the very time when general attention is thus being directed to some of the considerations arising out of defective health in early life the Education Committee of the London County Council will be called upon to recommend, and the Council itself to determine, on a question of great importance to the general efficiency of the elementary schools in many of the poorer portions of the metropolis. About six years ago the London School Board was rendered cognisant of the dirty and neglected condition of many of the children in their schools, and investigation by a committee showed grave reason for dissatisfaction. It became manifest, to many members of the Board, that the evils brought to light could best be remedied by submitting the children to regular inspection by trained observers; and, as it did not appear to be any part of the duty of the Board to supply the staff necessary for this purpose, the business of doing so was undertaken by a small society formed for the purpose, and known as the "School Nurses' Society." A committee was formed, partly from within the School Board and partly from outside, under the chairmanship of Lord Stanley of Alderley, a modest subscription list was set on foot, the Countess of Aberdeen, Lady Windsor, and other ladies assisted in various ways, and the society ultimately found itself able to employ five nurses, by whom regular weekly visits were paid to 80 schools. The business of the nurses was to deal at the beginning with a vast number of seemingly small ailments, many of which, if neglected, were capable of development into more serious ones, to keep a sharp look out for the early symptoms of epidemic disease and to urge promptitude in obtaining medical advice for them, to interview parents and explain to them the importance of cleanliness and the methods of obtaining it, to dress wounds, bruises, broken chilblains, sore eyes, and such like minor ailments, and to aim at the extirpation of vermin. Some of these matters might in themselves seem trifles, but they were like the trifles of the great sculptor in the well-known story. The consequences of attention to them were not trifling, and served in a very pronounced manner to raise the standards of the schools in which the nurses were employed. The teachers afforded hearty co-operation, and the parents, as a rule, were grateful for the advice of the nurses and made endeavours to act upon it. The benefits of the system became so apparent that the Board itself made a departure from its original decision on the subject, and appointed "experimentally," for a year, three nurses who have been engaged in carrying out, in selected schools, an elaborate and very successful scheme for the enforcement of personal cleanliness. It is obvious that a large part of the success, whether of the Society or of the Board nurses, would be dependent on the fact that both alike, in the former case indirectly, in the latter directly, were backed by the authority of the Board; and that the work which has been so well commenced cannot be continued unless the County Council will either undertake it, or will give to the School Nurses' Society a thorough and effective support. We cannot but think that the advantages of a competent sanitary supervision of the schools are so great and so manifest that these advantages ought to be supplied as part of the duty of the controlling authority, and that the Council will be well advised in brushing aside any difficulties which may stand in the way, and in continuing, in an effective manner and on a sufficient scale, the work which private charity has commenced of endeavouring to mitigate conditions by which the efficacy even of the best possible teaching must be in some degree impaired.

